# Environmental cadmium and breast cancer risk

**DOI:** 10.18632/aging.100226

**Published:** 2010-11-10

**Authors:** Carolyn M. Gallagher, John J. Chen, John S. Kovach

**Affiliations:** ^1^ Doctoral Program in Population Health and Clinical Outcomes Research, Stony Brook University, Stony Brook, NY 11794, USA; ^2^ Department of Preventive Medicine, Stony Brook University, New York, Department of Preventive Medicine, Z=8036, Level 3, HSC, Stony Brook University, Stony Brook, NY 11794, USA

**Keywords:** breast cancer, cadmium, NHANES, Long Island, New York

## Abstract

Breast cancer is the most prevalent women's cancer, with an age-adjusted incidence of 122.9 per 100,000 US women. Cadmium, a ubiquitous carcinogenic pollutant with multiple biological effects, has been reported to be associated with breast cancer in one US regional case-control study. We examined the association of breast cancer with urinary cadmium (UCd), in a case-control sample of women living on Long Island (LI), NY (100 with breast cancer and 98 without), a region with an especially high rate of breast cancer (142.7 per 100,000 in Suffolk County) and in a representative sample of US women (NHANES 1999-2008, 92 with breast cancer and 2,884 without). In a multivariable logistic model, both samples showed a significant trend for increased odds of breast cancer across increasing UCd quartiles (NHANES, p=0.039 and LI, p=0.023). Compared to those in the lowest quartile, LI women in the highest quartile had increased risk for breast cancer (OR=2.69; 95% CI=1.07, 6.78) and US women in the two highest quartiles had increased risk (OR=2.50; 95% CI=1.11, 5.63 and OR=2.22; 95% CI=.89, 5.52, respectively). Further research is warranted on the impact of environmental cadmium on breast cancer risk in specific populations and on identifying the underlying molecular mechanisms.

## INTRODUCTION

Breast cancer is the most prevalent women's cancer worldwide [1]. Although the rate of breast cancer in the United States, 122.9 per 100,000 U.S. women [2], is among the highest in the world [3], certain regions, including the northeastern states, have somewhat higher rates than the US overall. In particular, Suffolk County and Nassau County, Long Island (LI), New York (NY), with rates of 142.7 and 138.7 per 100,000 women from 2000-2004 [4] respectively, have been the focus of several studies looking for associations between environmental pollutants and breast cancer [5]. An increased risk of breast cancer was associated with home pesticide use [6], and residence within 1 mile of hazardous waste sites containing organochlorine [7]. Gammon [8] reported increased risk, without dose dependence, associated with polycyclic hydrocarbon adducts in DNA of circulating mononuclear cells, a measure of relatively recent exposure. A LI hospital-based study found no increased risk of breast cancer associated with organochlorine concentrations in breast adipose tissue [9] and Jacquez [10] found no association between airborne cadmium exposure and breast cancer rates on LI.

In 2006, McElroy [11] reported in a population based study that non-occupationally exposed Wisconsin women in the highest quartile of urinary cadmium (≥0.58 μg/g of creatinine), had twice the breast cancer risk compared to women in the lowest quartile (<0.26 ug/g) after adjustment for risk factors. Cadmium, a known carcinogen and risk factor for lung cancer [12,13], accumulates in the human body with age [14,15], and has several unique properties, including binding to and stimulation of the estrogen receptor alpha [16-18]and inhibition of DNA repair [19-22], which are potential risk factors for breast cancer carcinogenesis. Recently, Benbrahim-Tallaa [23] showed that cadmium directly transforms an estrogen receptor negative human breast cancer cell line incapable of xenograft formation to a basal-like breast cancer phenotype, which grows readily as a xenograft.

In humans, cadmium has an elimination half-life of 12 to 30 years [12, 24]. Cadmium is unique among the common heavy metal pollutants in that urinary cadmium is a biomarker of lifetime exposure, providing a means for assessing total body burden of cadmium[14,15]. Women tend to have higher cadmium levels than men presumably because of lower iron stores, which increase cadmium absorption [25,26]. Thus, comparable environmental exposures to cadmium may disproportionately affect women compared to men [25].

Because of the relatively high incidence of breast cancer on LI, we looked for associations between urine cadmium and breast cancer in a case-control sample of women living on LI obtained from the Long Island Database Project for Breast Cancer (LIDPBC), a repository of demographic and health data of female residents of LI. The results were compared to a cross-sectional U.S. probability sample, the National Health and Nutrition Examination Survey (NHANES 1999-2008) [27].

## RESULTS

In weighted, unadjusted, logistic regression analyses, log-transformed UCd, menopause, and continuous age in years were significantly associated with increased risk of breast cancer in both the NHANES (Table 1) and LIDPBC (Table 2) samples. The categorical variables for UCd quartile and age were significantly associated with increased risk of breast cancer in both samples. A dichotomous variable for below/above median UCd for LIDPBC controls was also significantly associated with increased breast cancer risk in the LIDPBC sample. Non-Hispanic white race was significantly associated with breast cancer in the NHANES sample. Smoking was significantly associated with breast cancer in the LIDPBC sample but not in the NHANES sample, and drinking was not significantly associated with breast cancer in either sample. Hormone use and later-/nulli-parity were not significantly associated with increased breast cancer risk in unadjusted analyses of either sample. LIDPBC cases had a statistically significant higher geometric mean of 0.58 μg cadmium/gram creatinine (μg/g) (median, 0.59) compared to 0.41 μg/g (median, 0.45) for controls. LIDPBC cases were older (63 years) than the controls (59 years) and fewer of the LIDPBC cases (43%) never smoked compared to LIDPBC controls (59%).

In a weighted, unadjusted, logistic regression model (Table 3), women in the highest cadmium quartile showed greater risk for breast cancer relative to those in the lowest cadmium quartile for both LIDPBC (OR=3.54; 95% CI=1.49, 8.42; p=0.004) and NHANES (OR=3.39; 95% CI=1.64, 7.05; p=0.001). Women in the third highest quartile showed significantly elevated odds for breast cancer in the NHANES sample (3.84; 95% CI=1.95, 7.55; p<0.001) and borderline association in the LIDPBC sample (OR=2.27; 95% CI=0.97, 5.33; p=0.060). The p value for trend of increasing risk with increasing urine cadmium quartile was significant for both samples. Age-adjustment attenuated this association but the trends for increased odds of breast cancer across increasing UCd quartiles remained statistically significant in both samples.

In a common, multivariable-adjusted model (Table 3) (adjusted for age group, smoking, drinking and menopausal status) for the NHANES sample, odds for breast cancer were significant and elevated for the third UCd quartile (OR=2.50; 95% CI=1.11, 5.63; p=0.028) and marginally significant for the fourth quartile (OR=2.22; 95% CI=0.89, 5.52; p=0.086). In the common LIDPBC model, the fourth quartile showed elevated odds for breast cancer relative to the lowest quartile (OR=2.69; 95% CI=1.07, 6.78; p=0.036). The trend for increased odds of breast cancer was significant across increasing UCd quartiles for NHANES (p=0.039) and LIDPBC (p=0.023). Adjustment for race/ethnicity in the NHANES model and for family history of breast cancer in the LIDPBC model did not alter these findings.

The LI never-smoker cases (n=43) and never-smoker controls (n=58) had significantly higher UCd, 0.53 ug/g and 0.46 ug/g, respectively, than the NHANES never-smoker cases (n=55), 0.38 ug/g and the NHANES never-smoker controls (n=1885), 0.33 ug/g (p=0.011 and 0.002, respectively). When smokers are included, the LI cases (n=100) also had a significantly higher median UCd, 0.59 ug/g, compared to the NHANES cases (n=99), 0.45 ug/g (p=0.003), whereas the median UCd of the LI controls (0.45 ug/g, n=98) was not significantly higher than the NHANES controls (0.39 ug/g, n=3120) (p=0.224).

**Table 1.** Characteristics of women age 30 years and older from NHANES (1999-2008) with p-values for simple logistic regression analysis for associations between independent variables and breast cancer

	Cases (n=99)	Non-cases (n=3,120)	Total sample (n=3,219)	p-value
UCd (μg/g) Geometric mean^a^ (std. dev.)	0.46 (.05)	0.39 (.01)	0.39 (.01)	<0.001
UCd (μg/g) Median	0.45	0.39	0.39	-
UCd Quartile^b^ # (%):				<0.001
Q1: UCd<0.22 (reference group)	10 (10%)	694 (22%)	704 (22%)	
Q2: 0.22≤UCd<0.37	24 (24%)	776 (25%)	800 (25%)	
Q3: 0.37≤UCd<0.60	34 (34%)	799 (26%)	833 (26%)	
Q4: UCd≥0.60	31 (31%)	851 (27%)	882 (27%)	
Age (years) Mean (std. dev.)	67 (13)	54 (16)	55 (16)	<0.001
Age group # (%):				<0.001
30≤years<55 (reference group)	21 (21%)	1,671 (54%)	1,692 (53%)	
55≤years<69	31 (31%)	733 (23%)	764 (24%)	
69 years and older	47 (47%)	716 (23%)	763 (24%)	
Never-smokers # (%)	55 (56%)	1,885 (60 %)	1,940 (60%)	0.763
Non-drinkers # (%)	37 (37%)	1,330 (43%)	1,367 (42%)	0.721
Non-Hispanic white # (%)	66 (67%)	1,534 (49%)	1,600 (50%)	<0.001
Ever used hormones^c^ # (%)	33 (36%)	845 (29%)	878 (30%)	0.435
Older than 26 years at 1^st^ live birth or nullipara^d^ # (%)	26 (28%)	619 (21%)	645 (22%)	0.465
Menopausal^e^^,^^f^ # (%)	84 (91%)	1,717 (60%)	1,801 (61%)	<0.001

Notes: Descriptive data unweighted; Simple logistic regression p-values generated using weighted data with statistical analysis for complex survey design

^a^ Urine cadmium values imputed for observations with value of zero by assigning ½* minimum detected value

^b^ UCd quartiles calculated based upon weighted creatinine adjusted cadmium frequencies for entire NHANES sample

^c^ NHANES sample includes 91 cases and 2,879 non-cases

^d^ NHANES sample includes 92 cases and 2,896 non-cases

^e^ NHANES sample includes 92 cases and 2,884 non-cases

^f^ Self-report of no period for 12 months in NHANES sample; excluded pregnant or lactating women from classification as menopausal.

**Table 2.** Comparison of characteristics for cases versus controls, women age 30 years and older from LIDPBC (2008-2009), with p-values for simple logistic regression analysis for associations between independent variables and breast cancer

	Cases (n=100)	Controls (n=98)	Total sample (n=198)	p-value
UCd (μg/g) Geometric mean^a^ (std. dev.)	0.58 (.05)	0.41^b^ (.05)	0.49 (.04)	0.001
UCd (μg/g) Median	0.59^b^	0.45	0.52	-
UCd Quartile^c^ # (%):	^b^			0.001
Q1: UCd<0.22	11 (11%)	23 (23%)	34 (17%)	
Q2: 0.22≤UCd<0.37	7 (7%)	14 (14%)	21 (11%)	
Q3: 0.37≤UCd<0.60	38 (38%)	35 (36%)	73 (37%)	
Q4: UCd≥0.60	44 (44%)	26 (27%)	70 (35%)	
LIDPBC UCd below and above control median^d^ # (%)	^b^			0.005
Below median (UCd≤0.40)	32 (32)	51 (52)	83 (42)	
Above median (UCd>0.40)	68 (68)	47 (48)	115 (58)	
Age (years) Mean (std. dev.)	63^b^ (9)	59 (11)	61 (10)	0.023
Age group # (%):				0.024
30≤years<55	17 (17%)	25 (26%)	42 (21%)	
55≤years<69	55 (55%)	58 (59%)	113 (57%)	
69 years and older	28 (28%)	15 (15%)	43 (22%)	
Never-smokers # (%)	43^b^ (43%)	58 (59 %)	101(51%)	0.023
Non-drinkers # (%)	26 (26%)	36 (37%)	62 (31%)	0.10
Non-Hispanic white # (%)	100^b^ (100%)	92 (94%)	192 (97%)	-^e^
Ever used hormones # (%)	35 (35%)	38 (39%)	73 (37%)	0.582
Older than 26 years at 1^st^ live birth or nullipara # (%)	61 (61%)	56 (57%)	117(59%)	0.58
Menopausal^f^ # (%)	89^b^ (89%)	64 (65%)	153 (77%)	<0.001

Note: Two-sample t-tests and Mann-Whitney U-statistics were used to compare central tendencies of cases versus controls, and chi-square and Fisher's exact tests (when applicable) were used to compare proportions of cases versus controls.

^a^ Urine cadmium values imputed for observations with value of zero by assigning ½*minimum detected value

^b^ Significant difference at α=0.05; LIDPBC cases compared to LIDPBC controls

^c^ UCd quartiles calculated based upon weighted creatinine adjusted cadmium frequencies for entire NHANES sample

^d^ LIDPBC UCd median cutoff point calculated based upon creatinine adjusted cadmium frequencies for LIDPBC controls

^e^ p-value was not calculated as cell frequency of 0 for non-white cases precluded logistic regression analysis

^f^ Self-report of no period for 6 months in LIDPBC sample; excluded pregnant or lactating women from classification as menopausal.

**Table 3.** Logistic model analysis results evaluating association between urine cadmium levels (UCd) (μg cadmium per g creatinine) and breast cancer in women age 30 years and older in NHANES (1999-2008, weighted) and LIDPBC (2008-2009) samples

	NHANES (1999-2008)		LIDPBC (2008-2009)	
UCd Quartiles	Odds ratio (95% CI)	p-value	Odds ratio (95% CI)	p-value
*I. Unadjusted model*				
	99 cases + 3,120 non-cases		100 cases + 98 controls	
Q1: UCd<0.22	1.00 (Reference)	-	1.00 (Reference)	-
Q2: 0.22≤UCd<0.37	1.85 (0.75, 4.56)	0.182	1.05 (0.33, 3.33)	0.940
Q3: 0.37≤UCd<0.60	3.84 (1.95, 7.55)	<0.001	2.27 (0.97, 5.33)	0.060
Q4: UCd≥0.60	3.39 (1.64, 7.05)	0.001	3.54 (1.49, 8.42)	0.004
p-value for trend test	<0.001		0.001	
*II. Age-adjusted model^a^*				
	99 cases + 3,120 non-cases		100 cases + 98 controls	
Q1: UCd<0.22	1.00 (Reference)	-	1.00 (Reference)	-
Q2: 0.22≤UCd<0.37	1.48 (0.59, 3.69)	0.399	1.01 (0.31, 3.32)	0.982
Q3: 0.37≤UCd<0.60	2.62 (1.21, 5.69)	0.015	2.23 (0.94, 5.28)	0.068
Q4: UCd≥0.60	2.09 (0.87, 5.00)	0.098	3.29 (1.36, 7.99)	0.008
p-value for trend test	0.039		0.004	
*III. Multivariable model adjusting for common variables*^b^				
	92 cases + 2,884 non-cases		100 cases + 98 controls	
Q1: UCd<0.22	1.00 (Reference)	-	1.00 (Reference)	-
Q2: 0.22≤UCd<0.37	1.34 (0.52, 3.45)	0.539	1.07 (0.31, 3.70)	0.910
Q3: 0.37≤UCd<0.60	2.50 (1.11, 5.63)	0.028	1.92 (0.77, 4.77)	0.162
Q4: UCd≥0.60	2.22 (0.89, 5.52)	0.086	2.69 (1.07, 6.78)	0.036
p-value for trend test	0.039		0.023	
*Model III, also adjusting for race*^c^*in NHANES and family history*^d^*in LIDPBC*				
Sample size:	92 cases + 2,884 non-cases		100 cases + 98 controls	
Q1: UCd<0.22	1.00 (Reference)	-	1.00 (Reference)	-
Q2: 0.22≤UCd<0.37	1.38 (0.54, 3.53)	0.507	0.98 (0.28, 3.46)	0.979
Q3: 0.37≤UCd<0.60	2.56 (1.13, 5.78)	0.024	2.01 (0.80, 5.04)	0.139
Q4: UCd≥0.60	2.32 (0.92, 5.84)	0.074	2.81 (1.11, 7.13)	0.030
p-value for trend test	0.034		0.017	

Notes:

NHANES model used weighted sample with Taylor linearization method for complex survey analysis; LIDPBC model used unweighted sample; Quartiles calculated based on weighted frequency distributions for NHANES entire sample

^a^ Adjusted for age groups 30-54, 55-68, 69+

^b^ Models include common independent variables that had a p<0.15 in the univariate analysis in either sample, including age group, never-smoker, never-drinker, menopausal status

^c^ NHANES model III also adjusted for race: non-Hispanic white relative to black, Hispanic or Mexican American, multi-racial or other

^d^ LIDPBC model III also adjusted for self-reported family history of breast cancer.

## DISCUSSION

Despite some differences between the LIDPBC and the NHANES samples with regard to race/ethnicity, age and lifestyle factors, the findings of both studies indicate an increased risk for breast cancer with increased body burden of cadmium, with similar effect estimates. These concordant findings from a cancer registry-based sample from a geographic region with a relatively high incidence of breast cancer and from a cross-sectional, national probability sample support an association between environmental cadmium exposure and risk of breast cancer, as initially reported in a non-occupationally exposed group of women in Wisconsin[11]. Since LIDPBC study is not probability-based, the results cannot be generalized to all LI women. The annual incidence of breast cancer is sufficiently rare, i.e., less than 1%(NYSDOH 2010), however, that the odds ratio can be used to estimate risk in our case-control study even though the control group is not a sample of the total population[28]. A sensitivity analysis using a dichotomous UCd variable (i.e., below and above median) based upon the UCd frequency distribution for LIDPBC controls showed similar results.

To estimate the potential impact of cadmium body burden on breast cancer risk for the exposed LIDPBC sample, we calculated an attributable risk percent for individuals with UCd ≥0.37 μg/g (weighted median for NHANES sample) based upon relative risk estimated from cross-tabulated frequencies for exposure and breast cancer in the LIDPBC sample. Because causality has not been established, we use the term excess fraction [28]. Assuming no unmeasured confounding, the estimated percent excess fraction for breast cancer among women with UCd ≥0.37 μg/g would be 43% for the LIDPBC sample. To take into account the complex survey design of the NHANES sample, and because NHANES is representative of the US population, we calculated population excess fraction for US women using the weighted, multivariable-adjusted odds ratio as a proxy for relative risk, which is appropriate given the rare disease assumption, e.g., 1% or less disease prevalence [28]. Based on a multivariable model, with binary UCd exposure (using a median cut) as a risk factor yielding a prevalence of 50%, the odds ratio was 2.0. Again, assuming no unmeasured confounders, 35% of breast cancer prevalence among US women would be attributable to cadmium exposures greater than or equal to 0.37 ug/g (95% CI=16%, 56%), an estimate similar to the population attributable risk estimated by McElroy [11] in a regional population study, i.e., 45 of 124 annual breast cancer cases per 100,000 women, or 36%. These estimates should be interpreted with caution, however, because the extent to which unmeasured confounders may have influenced results is uncertain.

A limitation of the NHANES sample is lack of family history of breast cancer. Additionally, self-report of breast cancer diagnosis might introduce a misclassification bias not present in the LIDPBC sample. Renal dysfunction may be induced by cadmium exposure [29] and by chemotherapy [30] but whether renal dysfunction affects cadmium excretion is uncertain. To evaluate whether renal dysfunction may have confounded our analysis of the association of urine cadmium with breast cancer, we added a covariate for self-report of physician diagnosed renal impairment to the NHANES model. There were only 3 participants who reported both renal impairment and breast cancer and inclusion of this covariate generated similar effect estimates for the association.

In the NHANES sample, the median difference between age at breast cancer diagnosis and age at UCd measurement was 7 years, and ranged from 5-12 years in the LIDPBC sample. Thus, women from both samples may have higher UCd levels than at the time they were diagnosed with breast cancer. To address a potential influence of incremental yearly age with regard to urine cadmium levels, a continuous variable for age in years was substituted for the categorical age group variable in each sample model, a continuous log-transformed UCd variable was substituted for UCd quartiles, and tested for interaction. The LI model showed a statistically significant association between breast cancer and the log-transformed UCd (OR=1.81, 95% CI=1.10, 2.96; p=0.019), but the interaction between age and UCd was not statistically significant and the model fit was adequate with main effects (Residual chi-square=0.655). The NHANES model, however, showed a statistically significant interaction (p=0.012). Stratification by median age (54 years) adjusted for year of age, showed that there was no significant association among women older than 54 years and significantly increased risk for women age 54 or younger in the third and fourth UCd quartiles (OR=6.35; 95% CI=1.05, 38.29; p=0.044 and OR=7.25; 95% CI=1.04, 50.72; p=0.046; p-value for trend test=0.010). Confidence intervals were wide due to small sample size. McElroy [11] also found cadmium-associated risk for breast cancer in younger women. Larger longitudinal studies are needed to evaluate the effects of UCd exposures at different ages on breast cancer risk.

The extent to which urine cadmium reflects long-term cadmium exposure may be influenced by age. Lauwerys [31] suggested that, at general environmental exposures, urinary cadmium levels parallel cadmium body burden until age 50-60 years but the extent to which recent exposures may also be reflected in urine cadmium among older general populations is uncertain. A possible limitation of both the NHANES and LIDPBC studies is the use of spot urine samples, which may constrain the accuracy of exposure assessment due to variable urinary dilution effects throughout the day [32]. Berlin [33], however, reported an excellent correlation between cadmium levels measured in spot and 24 hour samples from occupationally exposed subjects and spot urines have been used for numerous studies of lifetime cadmium exposure [34].

Breast cancer phenotype is another unmeasured factor, as cadmium has been associated with basal breast cancer cell transformation in tissue culture [23], and basal breast cancer is more common in younger women [35]. The frequency of basal cell breast cancer is about 15%, however, making it unlikely that our data in either sample is skewed because of a preferential induction of this type of breast cancer. A relatively longer time lag between breast cancer diagnosis and UCd measurement among NHANES participants could result in loss of subjects due to breast cancer mortality and underestimation of risk in that population. The cross-sectional design of the NHANES analysis limits interpretations of causality but, because UCd is a biomarker of lifetime exposure[12]), it is reasonable to assume that exposure to cadmium occurred prior to breast cancer diagnosis.

Although smoking is a well-established source of cadmium exposure, we found similar estimates for an association between increased UCd and breast cancer, independent of tobacco use in both samples, as was also reported by McElroy [11] in a Wisconsin regional sample. Additionally, in fully-adjusted Models III for NHANES and LIDPBC, the smoking covariate was not significantly associated with increased breast cancer risk in either sample. In consideration of cigarettes as a source of cadmium exposure, as well as inconsistent scientific findings regarding the association of breast cancer with smoking [36,37], we tested for interaction effects between smoking and the continuous cadmium exposure variable in multivariable analysis and the interaction term was not statistically significant in either the NHANES or the LIDPBC models.

The reason that never-smoking LI residents with or without breast cancer participating in this study have significantly higher total body burdens of cadmium than non-smoking women in the US sample is not known. Furthermore, a larger population-based study of breast cancer as a function of cadmium body burden in long-term residents of LI is needed to determine whether there is greater cadmium exposure in this region than in the US in general. If true, this could be a lead toward understanding at least one factor that may contribute to increased breast cancer risk in certain populations.

The molecular mechanisms underlying the ability of cadmium to increase risk of specific cancers remain to be delineated. The fact that cadmium may directly lead to cellular transformation of breast cells to a cancer phenotype and also bind to and activate the estrogen receptor alpha, as well as accumulate in breast adipose tissue, as reported by Antila [38], are intriguing leads to its association with breast cancer risk. Understanding the apparent multiple effects of cadmium is particularly challenging because of its ability to increase basal levels of oxidative DNA damage and to inhibit DNA repair [22,39,40]. Thus, Schwerdtle [22] showed in vitro that both water soluble and particulate cadmium disrupt nucleotide excision repair of bulky DNA adducts and UVC-induce DNA photolesions, supporting the hypothesis that cadmium acts as an indirect genotoxic agent.

The carcinogenic effects of cadmium could also be mediated in part by its ability to interfere with the function of p53, a key regulator of many components of DNA-damage induced defense mechanisms [41] and by its ability to substitute for zinc in proteins essential to cell integrity, such as XPA, an enzyme critical to nucleotide excision repair [42,43]. Kopera [44] showed that a peptide synthesized to resemble the zinc-finger domain of human XPA had a 1000-fold higher binding constant for cadmium compared to zinc and Asmuss [45] demonstrated that cadmium impairment of DNA-binding of purified XPA is reversed by zinc addition.

In addition, cadmium alters a number of molecular pathways, which regulate cell development and growth including the E-cadherin/β-catenin complex and genes activated in response to mitogens such as c-fos, c-jun, and c-myc and/or are induced by stress [46,47]. Chen [48] reported that cadmium induces multiple mitogen activated kinases and also activates mTOR, the mammalian target of rapamycin and that the clinically used drug rapamycin blocks cadmium-induced activity of mTOR, suppressing the cadmium-induced apoptosis of neural cell lines. Rapamycin has been shown to increase the lifespan of mice, mimicking the effect of caloric restriction, which is known to reduce mTOR activity and to increase the lifespan of many species. Anisimov [49] have recently shown that rapamycin increases lifespan and suppresses tumor development in cancer prone mice. Whether pharmacologic interventions to block or minimize the molecular actions of cadmium underlying its carcinogenic contributions are feasible are unknown but approachable challenges.

Our findings, like those of McElroy et al [11], indicate increased risk for breast cancer associated with increased urine cadmium concentrations that is independent of tobacco use. Although smoking is a well-established source of cadmium exposure, the major route of cadmium exposure is ingestion of food, particularly root vegetables, potatoes, and grain, including rice and wheat, grown on cadmium rich soils, and shellfish [50-56]. The estimated daily intake of cadmium in food in a non-hazardous environment for heavy metals is between 8 to 25 μg/day whereas one pack of cigarettes is estimated to add 1 μg/day[15,55]. Recently, phosphate fertilizers were cited by the President's Cancer Panel as a major source of cadmium in the food supply [56]. Cadmium enters the soil through the application of cadmium containing chemical fertilizers, municipal sewage sludge, and contaminated irrigation water to agricultural fields and worldwide through atmospheric deposition from gases emitted from industry. Important sources of cadmium pollution are byproducts of welding, electroplating, zinc and lead mining, smelting, disposal of nickel/cadmium batteries, and the plastics and pigment industries [55-56].

In addition to breast and other cancers [13,57], there is considerable evidence that cadmium contributes significantly to several common serious diseases in addition to cancers including osteoporosis [58-64]; stroke and heart failure [65], and renal tubular damage [15,34]. In 2009, the European Commission on Cadmium in Food lowered the permissible tolerable weekly intake of dietary cadmium from 7 ug/kg body weight (b.w.) per week to 2.5 ug/kg/ b.w. per week. This guideline was designed to keep the UCd below 1.0 ug/g in 95% of the population by age 50 years [55].The study of McElroy [11] and our present study, however, indicate that UCd below 1.0 ug/g is associated with increased risk of breast cancer.

Further research is needed to evaluate the extent of risk that increasing environmental concentrations of cadmium pose for breast cancer and to identify specific sources of cadmium exposure, particularly in geographic areas with high breast cancer rates. Moreover, studies of the interplay between cadmium exposure via particular foodstuffs and gastrointestinal absorption[26] may provide insights as to ways to mitigate cadmium's potential deleterious effects. At present, however, public health measures to reduce exposure to environmental cadmium including 1) decreasing fertilizer cadmium content; 2) informing the public about cadmium risks and identifying excessively rich cadmium foodstuffs; and, 3) minimizing release of cadmium from metal processing industries and electronic trash disposal is the first line of defense against this ubiquitous pollutant.

## METHODS

### Ethics statement

This investigation has been conducted in accordance with the ethical standards and according to the Declaration of Helsinki and according to national and international guidelines and has been approved by the Committee on Research Involving Human Subjects at Stony Brook University, Stony Brook, NY, USA.

Data for the case-control study of LI women were obtained from the LIDPBC comprised of 605 women, age 30 years or older, living on LI for, at least, 5 consecutive years prior to entry on study, 373 of whom had histologically documented breast cancer diagnosed after January 1, 1998 through December 31, 2004 and 232 women in the same region without a history of breast cancer. Cases in the LIDPBC had been identified through the cancer registry of Stony Brook University Hospital and contacted by their primary physicians to determine interest in participating. The control group was a purposive sample recruited from the community through local ads, presentations at health fairs, fundraisers for breast cancer research, and word of mouth, primarily through the breast cancer participants. Those providing informed consent completed a demographic, occupational, and health questionnaire under the supervision of a trained interviewer, including family history of breast cancer, use of hormone therapy other than birth control pills, age at first live birth, and menopausal status, provided a blood sample, and agreed to be contacted as to their interest in participating in future studies. For the present cadmium study, enrollees in the LIDPBC were invited to participate by mail. The first 100 women who had had breast cancer and the first 100 women without breast cancer to agree to the study were selected. Participants completed an informed consent, which included permission to use their questionnaire information on file in the LIPDBC, and provided a single urine sample. From 2008-2009, urine was collected in a coded cadmium-free urine container and sent to ARUP Laboratories (Salt Lake City, UT) for measurement of urine cadmium by inductively coupled plasma-mass spectrometry (ICP-MS)and measurement of urine creatinine.

Data were obtained from NHANES, a cross-sectional, random household survey of the civilian population based on a probability sampling design [66] for survey years 1999-2008. All women age 30 years and older, whose urine cadmium concentration were measured in a single sample by ICP-MS at the Center for Disease Control (CDC) were initially included. Urine collection tubes and storage containers were pre-screened by the CDC for background contamination of cadmium (CDC 2010). Analyses were then limited to women with UCd levels ≤20 ng/mL (unadjusted for urine creatinine), as recommended by Whittemore [67] to exclude observations with UCd levels beyond an upper bound for plausible values in environmental exposures. As a result, one participant without breast cancer diagnosis was excluded. Participants provided answers to a reproductive questionnaire, which included use of hormones other than birth control pills, age at first live birth, and menopausal status. Breast cancer was determined by self-report of a physician diagnosis; family history of breast cancer was not available in the NHANES sample.

The NHANES and LIDPBC study samples were summarized by unweighted descriptive statistics: medians, means (arithmetic and geometric) and standard deviations for continuous variables, and frequencies and proportions for categorical variables. Quartiles for creatinine-adjusted UCd were generated using weighted frequency distributions for the entire NHANES sample and used to evaluate the relationship between cadmium exposure and breast cancer in both the NHANES and LIDPBC samples. The quartiles were: 1^st^ quartile (Q1), UCd<0.22; 2^nd^ quartile (Q2), 0.22≤UCd<0.37; 3^rd^ quartile (Q3), 0.37≤UCd≤0.60; 4^th^ quartile (Q4), UCd≥0.60. In addition, a dichotomous UCd variable, for values below and above the median UCd, was created for the LIDPBC sample using UCd frequency distributions for LIDPBC controls: UCd≤0.40 and UCd >0.40 μg/g. Three age groups were analyzed: 30-54; 55-68; and 69 and older. For the NHANES sample, Medical Examination Center subsample weights for participants with urine cadmium measurements and Taylor linearization methodology were used for analysis of the NHANES sample in accordance with complex survey design. For each variable in the two study samples, univariate logistic regression analysis was performed to evaluate its association with breast cancer status. The analyses were based on weighted data for NHANES sample and unweighted data for LIDPBC sample. For the LIDPBC sample, comparisons between the LIDPBC cases and controls were also made using two-sample t-tests for geometric mean of UCd and arithmetic mean of age, Mann-Whitney U-statistic for median of UCd, and Chi-square and Fisher's exact tests (when applicable) for proportions, such as age group, smoking status, etc.

To evaluate the association between urinary cadmium and breast cancer, a series of logistic regression models were fitted to each sample including an unadjusted logistic model, an age-group adjusted logistic model, and a common logistic model, which adjusted for the same set of variables in both samples, and excluded variables with a p value >0.15 in univariate logistic regression analysis with breast cancer in both datasets. A final multivariable logistic model incorporating dataset-specific information, i.e., breast cancer family history, was also explored for the LIDPBC data, and race/ethnicity was added to the final NHANES model. Interactive effects between UCd and major covariates in association with breast cancer were also considered. We conducted further analysis of the sample stratified by the covariate, if the interaction term had a p-value ≤ 0.05. Odds ratios and their 95% confidence intervals (CI) were summarized. The linear association between UCd quartiles and breast cancer was analyzed by trend test. Models for complex survey design met the convergence criterion and case-control models met Hosmer-Lemeshow test for goodness of fit. Stata version 8.2 was used to perform t-tests. All other statistical analyses were performed using SAS version 9.2. A two-sided p value of less than 0.05 was regarded as statistically significant.
